# Tip Crack Imaging on Transparent Materials by Digital Holographic Microscopy

**DOI:** 10.3390/jimaging5100080

**Published:** 2019-10-01

**Authors:** Wen-Jing Zhou, Bo-Yu Li, Hong-Xia Shen, Deng-Ke He, Hong-Bo Zhang, Ying-Jie Yu, Vivi Tornari

**Affiliations:** 1 Department of Precision Mechanical Engineering, Shanghai University, Shanghai 200072, China; cistolie@shu.edu.cn (B.-Y.L.); vera_shx@shu.edu.cn (H.-X.S.); hedengkea@shu.edu.cn (D.-K.H.); yingjieyu@staff.shu.edu.cn (Y.-J.Y.); 2 Department of Computer and Information Sciences, Virginia Military Institute, Lexington, KY 24061, USA; zhangh@vmi.edu; 3 Institute of Electronic Structure and Laser, Foundation for Research and Technology, 71110 Heraklion, Greece; vivitor@iesl.forth.gr

**Keywords:** tip crack, transparent materials, digital holographic microscopy

## Abstract

With this study, we propose a method to image the tip crack on transparent materials by using digital holographic microscopy. More specifically, an optical system based on Mach–Zehnder interference along with an inverted microscopy (Olympus CKX53) was used to image the tip crack of Dammar Varnish transparent material under thermal excitation. A series of holograms were captured and reconstructed for the observation of the changes of the tip crack. The reconstructed holograms were also compared temporally to compute the temporal changes, showing the crack propagation phenomena. Results show that the Dammar Varnish is sensitive to the ambient temperature. Our research demonstrates that digital holographic microscopy is a promising technique for the detection of the fine tip crack and propagation in transparent materials.

## 1. Introduction

Fracture mechanics of crack propagation behavior under static and fatigue loads is an important subject. Over the past few years, mechanical failures have led to a growing awareness of the impact of cracks and stress in manufacturing parts on their failure strength. It is known that the crack bearing pressure or thermal load has an important effect on crack propagation. The number of cracks caused by environmental influences is significant; hence, by considering this relationship, the influence of pressure or thermal load on crack growth behavior can be evaluated by analyzing the stress intensity (or thermal) and the direction and number of crack growths [1,2]. P.F. Gao investigated the deformation in the fatigue crack tip plastic zone and its role in the fatigue crack propagation of a titanium alloy with a tri-modal microstructure by combining scanning electron microscopy and electron backscatter diffraction. It was found that the main crack changed by secondary microcracks [3]. Similarly, D. Nowell use in situ loading crack tips and observed their dynamics by using an electron microscope [4].

Digital holography is an optical tool that is used to reconstruct the phase and intensity maps of the tested objects simultaneously [5]. Digital Holographic Microscopy (DHM) presented in this paper takes advantage of both digital holography and microscopy with a Micro Objective (MO) in the object beam of the digital hologram recording system. Because the tested sample will be magnified through the MO, the resolution of the reconstructed information would reach a sub-micrometer level. DHM technique has been applied successfully in some fields such as Microelectromechanical Systems (MEMS) device morphology, deformation detection [6,7], workpiece surface roughness detection [8,9], non-preparation detection of living cells or biological tissue [10,11], transparent functionally gradient material detection [12], etc.

Notable applications of such examples include, for example, P. Asgari, who detected microstructural corrosion in austenitic stainless steel with a DHM, where the stainless steel temperature is increased until it is higher than the critical temperature; thereby, intergranular corrosion can be detected [13]. In another application, Shinichi Suzuki used high-speed holographic microscopy to take microscopic photographs instantly when the crack happens. It was found that the crack speed after bifurcation is slightly slower than prior to bifurcation [14].

With this research, we present a tip crack imaging [15] of the in-house made transparent sample by using DHM. The transparent sample was used to simulate the surface of a traditional oil painting. When the external temperature changes, the structure of precious oil paintings have a series of changes such as expansion, contraction, extension, and distortion. The varnish layer on the top of the painting is traditionally used to protect the painted surface materials [14]. However, the everyday cycling of temperature changes through the years results in an aesthetically disturbing cracking pattern on the top of painting surface. The cracks also expand, propagate, and deteriorate. The severity of the cracks is also influenced by other factors such as environmental temperature, humidity, and external vibrations [16,17,18]. It has been known that cracks either in varnish surface or in the constituted materials of the structure are of the most common problems in art conservation [19]. In our research, holograms of the tip crack were recorded with a regular time interval while the temperature continuously changed. The changes were detected by comparing several different reconstructed phases from the recorded holograms.

## 2. Key Principle of DHM

In off axis DHM, the reference beam R(x,y) is a plan wave, as follows:

(1) $$R\left(x,y\right)=A_{r}exp\left[i\varphi_{r}\right]exp\left[ik\left(t_{x}x,t_{y}y\right)\right]$$

where $A_{r}$ nd $\varphi_{r}$ are the amplitude and phase of the reference beam, respectively, and both are constants for uniform parallel laser beam. $t_{x}$, $t_{y}$ refer the tilt direction compared to the optical axial. k is wave number. The original object wave $O_{o}\left(x,y\right)$ after the plane beam being modulated by the tested sample and the MO is,

(2) $$O_{o}\left(x,y\right)=A_{z}\left(x,y\right)exp\left[i\varphi_{z}\left(x,y\right)\right]\exp\left[-\frac{ik}{2\mu }\left(x^{2}+y^{2}\right)\right]$$

where $A_{z}\left(x,y\right)$ and $\varphi_{z}\left(x,y\right)$ are the distribution of amplitude and phase modulated by the object, respectively. The quadratic term means that the spherical phase error is introduced by the MO, and μ is spherical phase curvature radius.

Hologram I(x,y) includes a zero-order image, the original objective wave, and the conjugate one [20] as follows:

(3) $$I\left(x,y\right)={\left|R\left(x,y\right)\right|}^{2}+{\left|O_{o}\left(x,y\right)\right|}^{2}+R^{*}\left(x,y\right)O_{o}\left(x,y\right)+R\left(x,y\right)O_{0}^{*}\left(x,y\right).$$

Here, * is a conjugate operation, and a Fourier spectrum window as high pass filter can be used to extract the original objective wave $R^{*}\left(x,y\right)O_{o}\left(x,y\right)$ [21],

(4) $$O_{z}\left(x,y\right)=R^{*}\left(x,y\right)O_{o}\left(x,y\right)=A_{r}A_{z}\left(x,y\right)exp\left\{i\left[\varphi_{z}\left(x,y\right)-\varphi_{r}\right]\right\}exp\left[-\frac{ik}{2\mu }\left(x^{2}+y^{2}\right)\right].$$

For numerical reconstruction of the digital hologram, the original objective wave should be recovered completely; hence, we need to simulate the reference beam R(x,y), and especially the compensation spherical phase in order to remove the spherical phase error ($exp\left[-\frac{ik}{2\mu }\left(x^{2}+y^{2}\right)\right]$ in Equation (4) as follows:

(5) $$O_{c}\left(x,y\right)=\exp\left[\frac{ik}{2\mu }\left(x^{2}+y^{2}\right)\right]$$

where μ is the spherical phase curvature radius that needs to be adjusted during numerical reconstruction. The object plane can be reconstructed by a convolution formula under paraxial approximation based on the Kirchhoff scalar diffraction formula [22] as follows:

(6) $$O_{r}\left(x,y\right)={ℱ}^{-1}\left[ℱ\left(R\left(x,y\right)\cdot O_{z}\left(x,y\right)\cdot O_{c}\left(x,y\right)\right)\cdot ℱ\left(g_{z}\left(x,y\right)\right)\right]$$

where ℱ is a fast Fourier transform operator. The impulse response function $g_{z}\left(x,y\right)$ of the convolution method is as follows:

(7) $$g_{z}\left(x,y\right)=\frac{i}{\lambda }\frac{exp\left[-j\frac{2\pi }{\lambda }\sqrt{x^{2}+y^{2}+z^{2}}\right]}{\sqrt{x^{2}+y^{2}+z^{2}}}$$

where λ is the wavelength of the laser source, and z is the distance between the object plane and the hologram plane. $O_{r}\left(x,y\right)$ is the reconstruction of the complex amplitude of the object wave including the intensity information and the phase information.

## 3. Preparation the Transparent Sample

**Figure 1:**
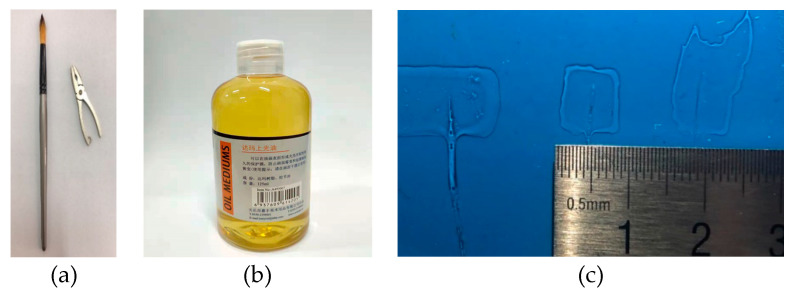
Experiment preparation. (**a**) Chinese writing brush. (**b**) Dammar Varnish. (**c**) Sample with a known defect.

We select a Chinese writing brush and Dammar Varnish, as shown in Figure 1a,b, to make the transparent sample with the original tip crack. The Dammar Varnish made of gum dammar and turpentine is often used to protect the paint by coating the surface of the paint. We pick up a piece of brush and put it on a clean piece of slide plate. Then the Dammar Varnish is used to paint evenly around the brush. Subsequently, we remove the brush and wait until the Dammar Varnish dries. In this way, a transparent sample with a known defect location can be obtained. As shown in Figure 1c, the area inside the red dotted rectangle is the test sample with a size of approximately 60 mm^2^. The cracks inside the white dotted ellipse are formed at the tip of the brush.

## 4. Experimental Setup

**Figure 2:**
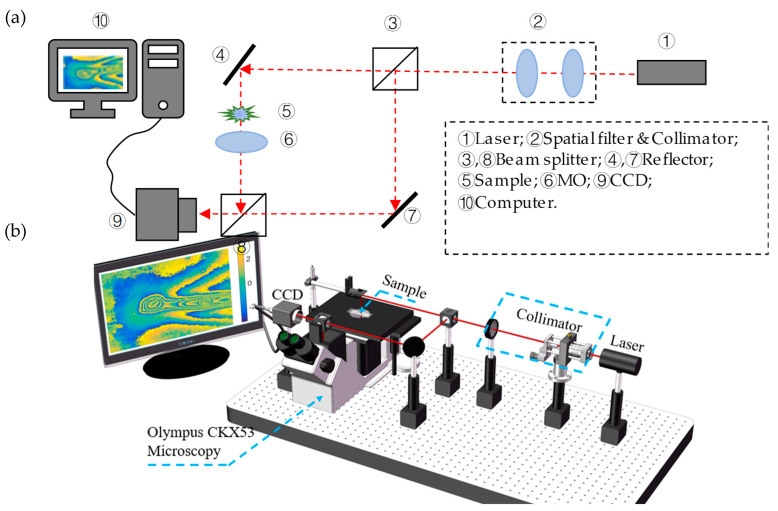
Experimental setup of Digital Holographic Microscopy (DHM) based on Mach–Zehnder interference with an inverted microscopy (Olympus CKX53). (**a**) Schematic diagram. (**b**) 3D sketch.

We set up an off-axis holographic optical system, shown in Figure 2a, which is based on the Mach–Zehnder interference system along with a MO to magnify the sample. As shown in Figure 2, a plane beam, after the He-Na Laser goes through the spatial filter and collimator, can be divided into objective and reference beams. Objective beam includes the diffractive wave of the magnified sample. The objective beam interferes with the reference beam on the hologram plane incident on the sensor plane of Charge Coupled Device (CCD).

A 3D sketch according to the experiment system is also given in Figure 2b. The Mach–Zehnder interference system was built based on an inverted microscopy (Olympus CKX53, Olympus, Shanghai, China). The MO has been set to 4X to magnify the sample. The He-Na Laser wavelength is 632.8 nm. DHC MER-500-7UM CCD (DAHENG, Shanghai, China) is used to record the hologram, and its size is 2592(H) × 1944(V) with a single pixel size of 2.2 μm × 2.2 μm. The distance from the image plane of the MO to the CCD is 28 mm, which is the recording distance of a hologram. Similarly, the reconstruction of the original image also takes place where the hologram is back and propagated to a distance of 28 mm.

**Figure 3:**
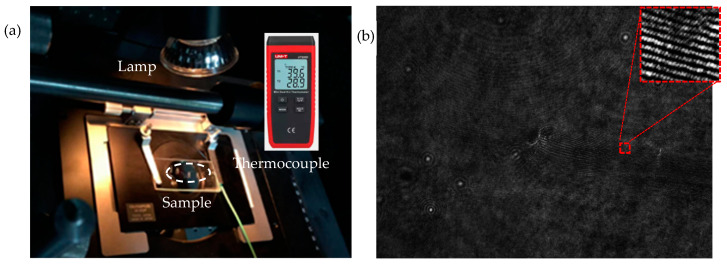
Photo of the heat loading and a representative hologram. (**a**) Photo of the heat loading. (**b**) Representative hologram.

Through the experiment, to stimulate the change of the tip crack, heat was used to illuminate the sample with a thermal 50 watts incandescent lamp. A thermocouple was used to monitor the change of temperature, as shown in Figure 3a. The precision was 0.1 degree centigrade and it was in direct contact with surface. The dotted circle was used to mark the tested area of the tip crack, as shown in Figure 3b. A hologram shown in Figure 3b was captured before the heat was applied. While the sample was heated, six holograms were captured every 4 h. The first step was a 12 h heating. The temperature increased quickly in the beginning and slowly later, and the 12 h heating was used to reach a balance that demonstrates that the temperature changes slowly. When we heated it for 24 h and the temperature was steady, we added 12 h of heating, and then more 4 h of heating. It took 40 h to complete the recording process of all holograms. The temperature reached between 36.5 °C and 38 °C. The heating time and the recorded temperature for each hologram are presented in Table 1.

On a specific note, for the prevention of the recorded hologram being influenced by the lamp, the lamp was turned off before the hologram was captured; hence, the temperature recorded here in Table 1 is the temperature before the lamp was turned off.

## 5. Experimental Results and Discussion

**Figure 4:**
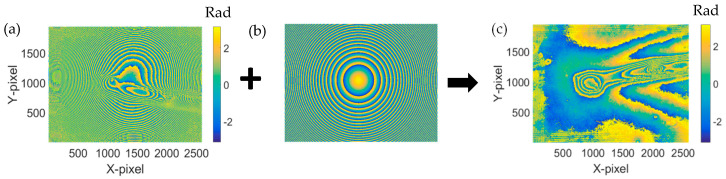
Reconstructed original phase distribution for reference hologram before heating. (**a**) With the spherical phase error. (**b**) Numerical compensation phase. (**c**) Phase distribution following compensation phase.

The hologram was reconstructed using the convolution algorithm [5]. The phase distribution is shown in Figure 4a. It is clear that there is a spherical phase error due to the use of 4X MO. A numerical compensation was used to remove such a spherical phase error, which is shown in Equations (5) and (6). The compensation phases shown in Figure 4b,c present the correction phase distribution, which is the wrapped phase information of the sample before being heated.

**Figure 5:**
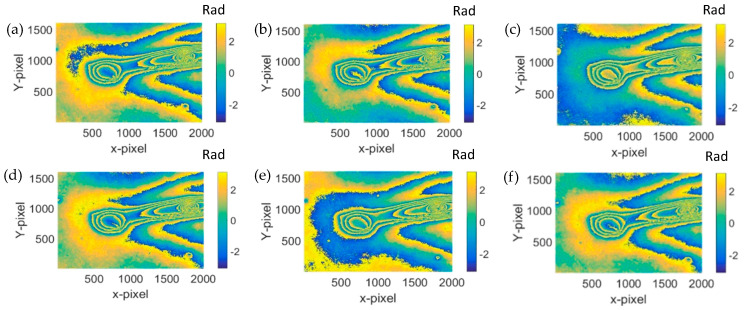
Reconstructed phase distributions after compensation of spherical wave for different heating time. (**a**) 12 h; (**b**) 16 h; (**c**) 20 h; (**d**) 24 h; (**e**) 36 h; and (**f**) 40 h.

**Figure 6:**
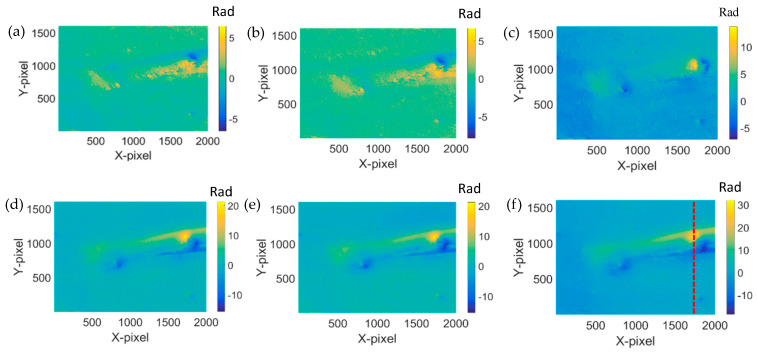
Phase difference after the reconstructed phase in Figure 5 minus the original phase in Figure 4c. (**a**) 12 h–0 h; (**b**) 16 h–0 h; (**c**) 20 h–0 h; (**d**) 24 h–0;. (**e**) 36 h–0 h; and (**f**) 40 h–0 h.

The reconstructed phase distribution after the sample being heated is shown in Figure 5. The differences between the phases in the consecutive phase distribution are clear, indicating the obvious. The thermal influences are due to lamp heating. It is suspected that such changes are due to the fact that the Dammar Varnish material coated on the glass plate was expanded due to the heat. However, new cracks did not occur through these phases. In order to observe the changes of the tip crack on the sample clearly, the phase unwrap was performed [5,23]. The unwrapped phase was also differentiated between the initial frame and the consecutive frames. Figure 6 presents the differentiated phase where the differences of the phases are clear.

**Figure 7:**
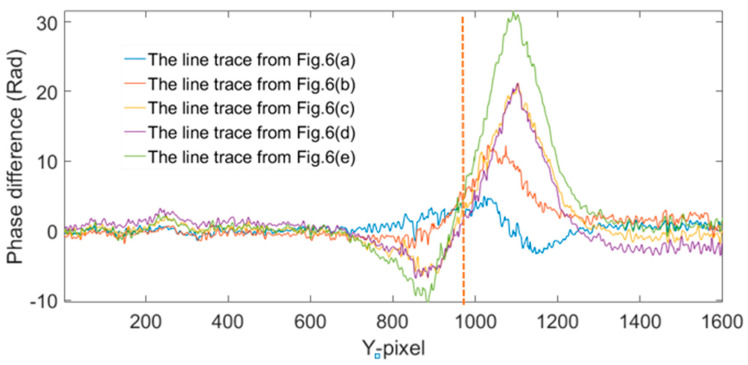
Six phase line traces extracted from each of phase difference in Figure 6a–f, respectively.

We have selected the most significant section of the phase in Figure 6 at the dashed line position, and have extracted and are showing them in Figure 7. It is clear that the phase increases while the heating time is increased. The minimum phase difference corresponds to the shortest heating time, while the maximum one represents the longest heating time.

As shown in Figure 7, on the right part of the dash line, the phase increases. It is therefore safe to conclude that the interspace of the crack has expanded because of heating. As a result, there is internal force caused by the expanded interspace. On the left of the dash line, the interspace should be compressed, which means the phase changes are decreased. Hence, the phase differences on the left of the dash line should correspond to the actual phase differences of the tip crack.

This result indicates that the cracking behavior was altered when it was exposed to the thermal effect induced by the thermal lamp. Hence, it shows that by the implementation of DHM, the visualization of cracks propagation is viable.

## 6. Comparison Experiment Setup and Results

**Figure 8:**
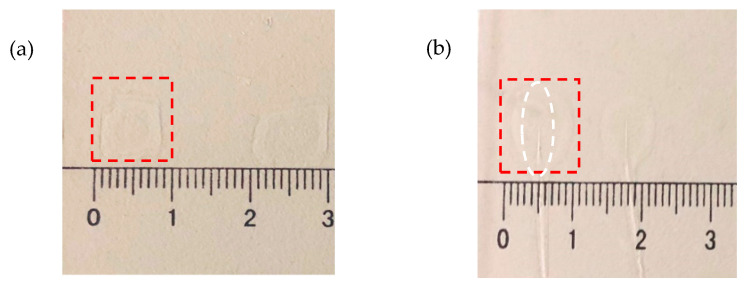
Samples used in contrast experiments: (**a**) sample without crack and (**b**) sample with crack.

A contrast experiment was also conducted to verify that the phase changes (Figure 7) are actually caused by a crack being heated rather than by other confounding factors. For this purpose, two new samples were prepared, as shown in Figure 8. One without a crack is shown in Figure 8a, and it is approximately 60 mm^2^, while the cracked one is shown in Figure 8b, and it is about 60–65 mm^2^. For comparison, we did two comparative experiments: the first sample without a crack was heated to observe whether the existed crack affected the phase change, and the second sample with a crack was no longer heated to observe whether heating affected the phase change. For both of samples, a serial of holograms was recorded for every four-hour period, the same as with the experimental group above. The heating time and the temperature of the two samples are listed in Table 2 and Table 3, respectively.

**Figure 9:**
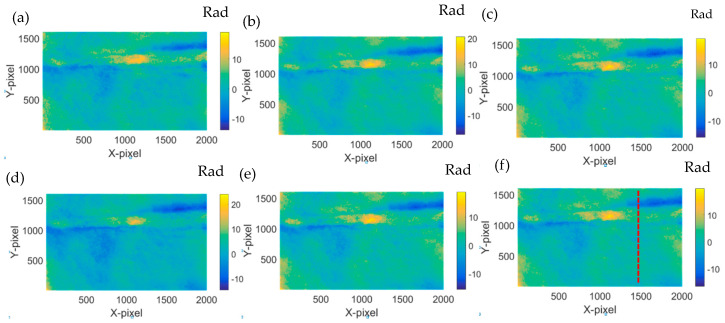
Phase difference after the reconstructed phase of the sample with crack and no heating. (**a**) 12 h–0 h: (**b**) 16 h–0 h: (**c**) 20 h–0 h: (**d**) 24 h–0 h: (**e**) 36 h–0 h: and (**f**) 40 h–0 h.

**Figure 10:**
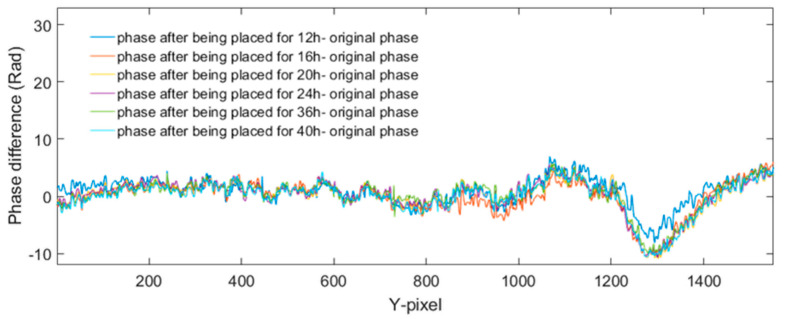
Six phase line trace extracted from each of phase difference of comparison experiment by a sample with crack and no heating.

**Figure 11:**
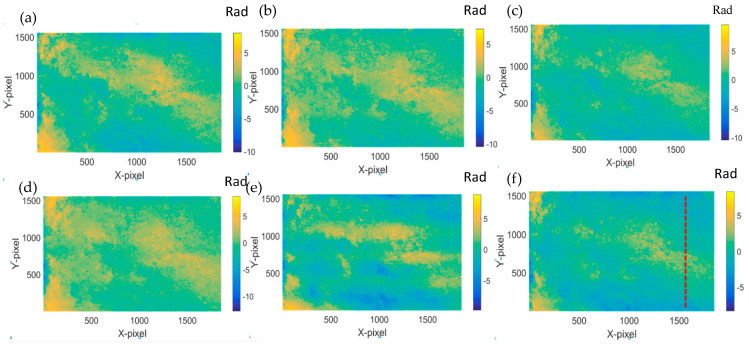
Phase difference after the reconstructed phase of the sample with no crack and heating. (**a**) 12 h–0 h; (**b**) 16 h–0 h; (**c**) 20 h–0 h; (**d**) 24 h–0 h; (**e**) 36 h–0 h; and (**f**) 40 h–0 h.

**Figure 12:**
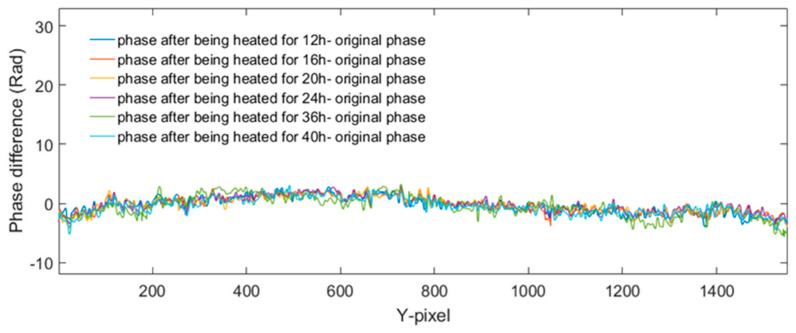
Six phase line trace extracted from each of phase difference of comparison experiment by a no-crack sample and heating.

Experimental results about the phase change based on the holograms obtained according to the values of Table 2 and Table 3 are shown in Figure 9, Figure 10, Figure 11 and Figure 12. The phase changes are calculated by the same method mentioned in the previous experiment. We found that the phase of both of the samples does not undergo changes. Hence, that means the crack changes of the tip cracks on transparent material are due to the changes of temperature rather than other factors.

## 7. Conclusions

With this study, the cracks and deterioration of transparent material were detected by DHM. The tip crack propagation of the material is clearly shown through the experimental results. Thus, the results support the notion that DHM is a viable candidate to detect the fine tip crack of some transparent materials. Detailed phase changes were shown, demonstrating that varnish material is sensitive to heat. The results show that in order to preserve the Dammar Varnish materials, the insulation of the materials from heat impact is necessary. The consequences of this work in cultural heritage can be multiple. First, it is proven that a DHM system is worth further investigation for its potential to be developed into a portable investigation system capable of being used indoors and outdoors. Secondly, it is a method worth studying further as a method that can be used to document with highest possible accuracy the existing crack condition of important artworks, and to regularly monitor any alterations. This offers not only a highest standard crack documentation method, but also an insight for conservation scientists into how the surface cracks deteriorate and how quickly, allowing them to evaluate the risk in each artwork separately by only the tip crack coordinates. In the future work, it would be interesting to use a tunable wavelength laser to detect the crack propagation of non-transparent material in cultural products.

## Figures and Tables

**Table 1:** The heating time and the recorded temperature for each hologram.

No. of Hologram	0	1	2	3	4	5	6
Heating time (h)	0	12	16	20	24	36	40
Temperature (°C)	22.3	36.5	36.8	36.8	38	37.1	37.8

**Table 2:** The heating time and the temperature at each hologram of the sample without a crack.

No. of Hologram	0	1	2	3	4	5	6
Heating time (h)	0	12	16	20	24	36	40
Temperature (°C)	20.0	34.7	36.4	37.3	37.6	37.0	37.8

**Table 3:** The heating time and the temperature at each hologram of the sample with a crack.

No. of Hologram	0	1	2	3	4	5	6
Heating time (h)	0	12	16	20	24	36	40
Temperature (°C)	19.3	19.2	19.5	20.0	19.3	18.8	20.1
